# A web-based knowledge database to provide evidence-based information to cancer patients: Utilization within the PIKKO study

**DOI:** 10.1007/s00520-024-08725-7

**Published:** 2024-07-17

**Authors:** Nico Schneider, Uwe Altmann, Florian Brandt, Jutta Hübner, Bernhard Strauss, Christian Keinki

**Affiliations:** 1 Institute of Psychosocial Medicine, Psychotherapy and Psychooncology (IPMPP), Jena University Hospital, Stoystrasse 3, 07740 Jena, Germany; 2 Department of Psychology, MSB Medical School Berlin GmbH, Berlin, Germany; 3 IKK Südwest, Europaallee 3-4, 66113 Saarbrücken, Germany; 4 Department of Haematology and Medical Oncology, Jena University Hospital, Jena, Germany; 5 The German Cancer Society, Berlin, Germany

**Keywords:** Cancer, e-health, Health literacy, Website, Patient navigator, Information, Quality

## Abstract

**Purpose:**

Cancer is associated with an urgent need for understandable and reliable information, which is often not satisfied by information available online. Therefore, as part of the PIKKO project, a web-based knowledge database (WDB) was introduced to provide cancer patients with quality-assured, evidence-based information. This paper aims to provide insights into the usage (Who? How? What?) and the effects regarding health literacy of the WDB.

**Methods:**

A patient survey and automatically generated logfile data were evaluated. Two user groups, patients and patient navigators (PNs), were compared.

**Results:**

The 13 PNs were responsible for 1/3 of all accesses over the entire duration of the project. The 413 patients used WDB twice on average and spent an average of 12 min per session online (PNs: 9 min per session, more frequently). The top 3 topics of interest were ‘therapy’, ‘nutrition’ and ‘carcinogenesis’ for the patients, and ‘therapy’, ‘naturopathy’ and ‘legal regulations/support’ for the PNs. Of the patients surveyed, 69% said that WDB was helpful in making informed decisions, 76% found the information they wanted and 90% thought WDB was an appropriate way to provide information.

**Conclusion:**

Our WDB provided important information about cancer and its treatment on a digital way both, to patients and PNs. In routine cancer care, the WDB can improve health literacy and informed decision-making.

**Trial registration:**

This study was retrospectively registered in the German Clinical Trial Register under DRKS00016703 (21 Feb 2019, retrospectively registered). https://www.drks.de/drks_web/navigate.do?navigationId=trial.HTML&TRIAL_ID=DRKS00016703

**Supplementary Information:**

The online version contains supplementary material available at 10.1007/s00520-024-08725-7.

## Introduction

Cancer is undoubtedly one of the most serious events in a person's life. Such events can be associated with anxiety, concerns, and important decisions. In surveys of cancer patients, they frequently expressed a desire for more information [1]. Information can help to reduce anxiety [2] and to make decisions more thoughtfully [3]. However, the need for information and the perceived lack of information depend, for example, on the socio-demographic situation and differ at every phase of cancer care trajectory [1, 4].

Nowadays, cancer patients' information needs are mainly met through direct conversations with medical staff, but the importance of online resources increases when searching for health information. While the internet was in third place a few years ago, it is now the second most common used source for information [5–8].

Information related to cancer available online is very heterogeneous. This information is often incomplete, of insufficient or poor quality, or difficult to read [9, 10]. The quality is highly dependent on the particular provider or website publisher, as studies on immunotherapy [11], bladder cancer [12], thyroid cancer [13], multiple myeloma [14], and radiotherapy for lung cancer [15], or breast cancer [16] showed. Furthermore, there are substantial differences between oncologists' and non-medical practitioners' websites [9].

Liebl et al. [17] analyzed 77 German websites with cancer information and found a discrepancy between visibility (which sites appear at the top of online searches) and quality criteria such as the HONcode [18]. Herth et al. [10] analyzed 60 German websites regarding cancer diets and found significant differences between many profit-driven websites with lower quality and only a few trustworthy websites with higher quality. On the one hand, when searching for information, patients and people who advise or care for patients, have many opportunities and possibilities through the internet. On the other hand, patients and their advisors or caregivers are also increasingly faced with challenges mainly regarding the flood of information and the quality of the information provided. When using the Internet (e)health literacy skills are becoming increasingly important.

Providing cancer patients with quality-assured, evidence-based information, which are readable and understandable for medical laypeople, was one of the goals of the PIKKO project [19]. A web-based knowledge database (WDB), which could be accessed via a website, was one module in this project. Patients should be able to find reliable information about their disease in one trustworthy place in order to improve individual health literacy and to support informed decisions during the decision making process. For the same reasons, this WDB should also be available to patient navigators, who should advise and support patients.

This paper aims to provide information about the utilization of a patient-oriented, web-based information database and therefore considers the following questions: Who were the users of the WDB? How was the WDB used? Which content was used? How did users rate the WDB? Furthermore, the influence of the usage of the WDB on the health literacy of the participating patients in PIKKO will be described.

## Methods

The STROBE Statement for cohort studies 2007 [20] was used to report this study.

### Setting

This analysis was part of the project “Patient information, communication and competence empowerment in oncology” (PIKKO).

#### The PIKKO project

PIKKO was a care concept for cancer patients. It was funded by the Innovation Fund of the Federal Joint Committee in Germany from 2017 to 2021 (funding number: 01NVF17011). It provided cancer patients with an additional counseling and information pathway that consisted of the modules (1) patient navigator (PN) for first level support around oncological care, (2) courses (e.g., nutrition, physical activity) as well as psychological and psycho-social counseling service offered by the Saarland Cancer Society, and (3) a web-based knowledge database (WDB). In the PIKKO project [19], an intervention group (IG) that had access to all three modules was compared with a control group (CG) without access to the modules. Data on the use of the intervention modules and various psychological parameters, including health literacy, were analyzed. Patients were surveyed up to five times (baseline + one follow-up every three months).

#### Web-based knowledge database

The WDB named "My PIKKO" was developed and provided by the German Cancer Society (GCS) for the PIKKO study. It was accessible via the internet from 2018 to 2020. Each patient in the IG was given an individual login at the initial appointment with the PN through which the WDB could be used as often and as long as they desired. Information on patient name, date of birth, and cancer type was stored securely in terms of data protection law and was not part of the analysis.

The WDB consisted of several chapters: general information on cancer, information on the patient's specific type of cancer (e.g., breast cancer), and socio-legal topics. Cancer chapters included topics on carcinogenesis, prevention, diagnosis, therapy, nutrition, physical activity, psychological support, palliative care, side effects, and naturopathy. Socio-legal topics included insurance, legal regulations and support, rehabilitation, and financial matters. In addition, there were checklists and guidance notes, the possibility to search for relevant addresses, a reading list, a glossary for medical vocabulary, and a list of abbreviations.

The texts were written in a way that was understandable to laypersons, using quality-assured, evidence-based information provided by GCS staff, and were updated throughout the duration of the project. The evidence base was ensured through systematic literature searches in MEDLINE and EMBASE (via Ovid). Since the evidence base was generally reduced in the case of rare tumors, secondary literature was also used in such cases, for example Clincal Practice Giudelines by the ESMO [21]. After the texts were created, a comparison was made with the German guidelines [22]. Since the texts were primarily intended for medical laypeople, criteria of quality-assured health information were applied (in particular “DISCERN” [23], “HONCode” [18, 24] and “Good Practice Health Information” [25]). Overall, the texts were written in a neutral language; aggravations or trivializations were not used, and no interpretations of the medical information or recommendations for action were described. With regard to readability, the texts were written using the Flesch-Reading Ease Scale [26] and the fourth Vienna Formula (in German: Wiener Sachtextformel) [27] to create texts with an average readability level of 9 to 10 years of schooling. The formulas were incorporated in the editorial backend so that the readability of a text was automatically shown and could be used in real-time by the GCS staff.

In addition to patients, PNs also had their own login. They could use this to inform for themselves and to search for information together with the patient during an appointment or to demonstrate the use of the WDB.

Two screenshots of the WDB can be seen in online resource 1. To view a demo version of the WDB (in German, for other languages, please use the translation algorithms integrated into the browser), please follow the link in the reference and use the word ‘testnutzer’ as username and password [28].

### Study design

The analysis of the use of the WDB was done in an observational study. Access to the database by persons authorized to do so was recorded over a period of two years, including the time of access and database pages visited. Two cohorts were investigated: patients and PNs.

### Participants

As described, all IG patients and all PNs had access to the WDB. However, the usage was voluntary. Patients in the IG met the eligibility criteria of the PIKKO study [19], i.e. age between 18 and 90 years, any cancer of any stage, no statutory guardianship, sufficient knowledge of the German language, no severe visual or hearing impairment, no dementia or other mental limitations. PNs were medical assistants or nurses with at least two years of professional experience in the oncology field. They received special training by the GCS in using PIKKO to provide support and counseling to patients.

### Variables

To evaluate the utilization of the WDB, various parameters were compared between the groups (IG, PNs):

- number of users (absolute number and percentage)
- accesses to the WDB (absolute number and percentage)
- accesses per single user (mean, M, standard deviation, SD, maximum, max, and median)
- accesses per day per single user (M, SD, max and median)
- the total time spent on the WDB per individual user in minutes
- the total time per individual user per access in minutes
- one-time accesses as well as accesses over different months (1 month, 3 months, …, 12 months, more than 12 months) (absolute number and percentage)
- number of patients who accessed the topic (absolute number and percentage)
- accesses to the topic (absolute number and percentage)
- the median time spent on the topic in seconds

In addition, to clarify the acceptance of the WDB among patients, responses to the following questions were collected (Table 1):

**Table 1:** Additional questions as part of the PIKKO survey on the web-based knowledge database (WDB) regarding usage

Question	Answer options
If you did not use the WDB, why not?	open-ended text
What did you use the WDB for?	a choice of answer options
Do you usually use the internet to get information?	yes/no
Did "My PIKKO" help you make more informed decisions?	yes/no
Have you been able to find all the information you needed?	yes/no
Do you think "My PIKKO" is an appropriate way to provide information about diseases?	yes/no
What suggestions do you have for improvement and what information or functions did you miss?	open-ended text

For multiple-choice or yes/no questions, the frequency of the different responses (absolute number and percentage) were reported. Lastly, an overall patient rating was reported, which is a 6-point scale modulated after the German school grading system with which each participant was familiar (1 = very good to 6 = insufficient).

### Data sources

All variables of utilization were taken from the logfile of the WDB. This file was read out by the technical administrator at the end of the data collection in the PIKKO project on October 3, 2020. In the logfile, every access to a subpage was recorded. The subpage was archived with its ID, title, topic, and chapter affiliation. In addition, each access was assigned to the user group. Since the transfer of knowledge was the goal of the WDB, only visits on the content elements were tracked. The use of the checklists, guidance notes, address search, reading lists, glossary and list of abbreviations were not recorded.

From the PIKKO patient survey, the total number of patients who had potentially gained access to the WDB (= all patients with an initial appointment with PN, regardless of their active participation in the intervention and/or patient survey) was determined. The total number of PNs participating in the PIKKO study, as well as socio-demographic and clinical characteristics of participating patients and answers to the patient acceptance questions were also taken from this source [19].

The analysis of the influence of the WDB on health literacy has already been described by the authors elsewhere [29] and will be taken up here due to its relevance in terms of content. For a description of the statistics of this analysis, please also refer to this publication.

### Sample size

753 patients were included in the IG in the PIKKO project [19].

### Quantitative variables

The variables recorded in the logfile could be used without much modification. The time a user spent on a page was calculated based on the timestamp when the page was accessed. The duration is crucial for all variables that are related to time.

#### Health literacy

Health literacy was measured as part of the PIKKO survey [29] at all measurement time points using the HLS-EU-Q47 [30] (follow-up measurements: n = 301, min = 1, max = 4, median = 2). The score ranges from 0 to 50, with higher values indicating a higher level of health literacy. The cut-off between limited and non-limited health literacy is 33.

### Analyzes of qualitative variables

Patients' open-ended explanations for not using the WDB (from the patient survey) were grouped into six categories: *technical reasons*, *no need*, *no time*, *avoidance of thinking about cancer*, *health reasons*, or *no motivation*.

All suggestions for improvement or missing information and functions were assigned to one of six categories: (1) *wish for more information*; (2) *wish for structural or design changes*; (3) *wish for interactivity with medical staff*; (4) *wish for the possibility of exchanges with other patients*; (5) *suggestions for further functionalities*; (6) *wish for functions regarding doctor appointments*.

Both category systems were formed by inductive coding [31]. All statements could be clearly assigned to the categories mentioned.

### Statistical methods

To examine differences between users and non-users of the WDB in terms of their socio-demographic and clinical characteristics, F-tests and chi-square test were performed (independent variable: user or non-user; dependent variables: age, gender etc.). Cramer-V [32] (V > 0.1: small effect; V > 0.3: medium effect; V > 0.5: strong effect) and partial Eta-squared [33] (ƞ^2^ > 0.01: small effect; ƞ ^2^ > 0.06: medium effect; ƞ ^2^ > 0.14: strong effect) were used as effect sizes.

User numbers, access data and patient acceptance questions were analyzed descriptively.

To analyze the context, we used the accessed subpages that were already categorized as topics on the website. Then we determined which topics were accessed how often by the two groups. For each topic, the absolute and relative frequencies of accesses, the absolute and relative number of patients who accessed the topic, and the median time spent (in seconds) on each topic, were determined. The variables were then ranked to show which topics were of most interest to patients and PNs.

The software we used was SPSS Statistics 27.

## Results

### Participants

**Fig. 1:**
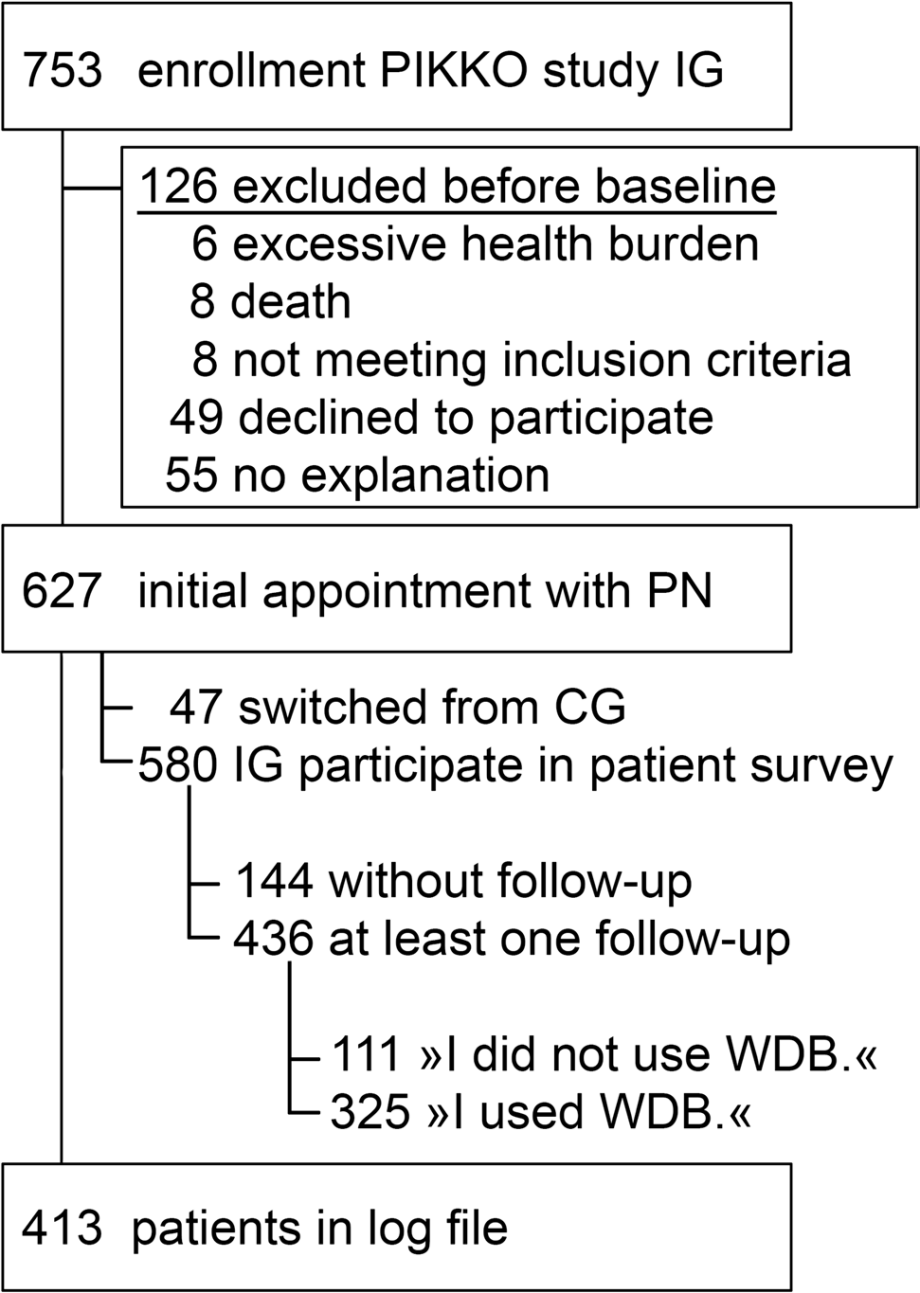
This flow chart combines numbers from the patient survey (participant intervention group, IG) and from the logfile (user group *patients*)

A total of n = 15 PNs worked in the PIKKO project. According to the logfile, 86.7% (13/15) used their own login. For the IG of the PIKKO project, 753 cancer patients were recruited. Of these, 110 dropped out prior to initial meeting with the PNs, eight were excluded due to missing eligibility criteria, and eight died (see Fig. 1). This left 83.3% (627/753) who could potentially have received a login to the WDB in an initial appointment with the PN. According to the logfile, 65.9% (413/627) used their login at least once to access the WDB.

In the patient survey, n = 580 patients in the IG participated at baseline. In total, 75.2% (436/580) answered questions about the WDB across all follow-ups. Of these, 25.5% (111/436) stated that they never used the WDB and 74.5% (325/436) used it at least once. See also Fig. 1.

The reasons for never using the WDB were: 25.2% (28/111) for technical reasons, 24.3% (27/111) no need, 13.5% (15/111) no time, 6.3% (7/111) avoid of thinking about cancer, 5.4% (6/111) for health reasons, and 4.5% (5/111) no motivation. In n = 37 cases, no reason was given.

### Descriptive data

#### Patients

Characteristics within the subgroup of users are presented and compared with the non-users in Table 2.

**Table 2:** Baseline socio-demographic and clinical characteristics of users and non-users of the WDB

	user	non-user	statistic
Number of patients	325	111	
Age *M (SD)*	57.8 (10.0)	58.8 (12.3)	F(1, 434) = 0.662, p = 0.416, η^2^ = 0.002
Gender %* (n)*			
Female	70.2% (228)	61.3% (68)	χ^2^(1) = 3.0001, p = 0.083, V = 0.083
Male	29.8% (97)	38.7% (43)	
School level %* (n)*			
< 10 years of school	43.1% (140)	56.8% (63)	*χ*^*2*^*(2)* = *6.612, p* = *0.037, V* = *0.123*
10 years of school	28.9% (94)	24.3% (27)	
> 10 years of school	28.0% (91)	18.9% (21)	
Education years (school + vocational) *M (SD)*	12.6 (3.1)	11.8 (2.9)	*F(1, 434)* = *5.952, p* = *0.015, η*^*2*^ = *0.014*
Marital status %* (n)*			
Single	13.5% (44)	15.3% (17)	χ^2^(3) = 1.701, p = 0.637, V = 0.062
Married	69.5% (226)	64.9% (72)	
Divorced	11.1% (36)	10.8% (12)	
Widowed	5.8% (19)	9.0% (10)	
Living with the partner %* (n)*	78.1% (250)	72.5% (79)	χ^2^(1) = 1.451, p = 0.228, V = 0.058
Children living in the household %* (n)*	22.0% (70)	20.7% (23)	χ^2^(1) = 0.081, p = 0.776, V = 0.014
Financial worries %* (n)*	12.4% (40)	16.4% (18)	χ^2^(1) = 1.120, p = 0.290, V = 0.051
Period of the most recent illness %* (n)*			
up to 1 year (acute)	78.4% (243)	80.0% (84)	χ^2^(2) = 0.204, p = 0.903, V = 0.022
2–5 years	16.1% (50)	14.3% (15)	
> 6 years	5.5% (17)	5.7% (6)	
Enrollment inpatient %* (n)*	77.2% (251)	64.9% (72)	*χ*^*2*^*(1)* = *6.590, p* = *0.010, V* = *0.123*
Groups of cancer %* (n)*			
Gastrointestinal (C00-25)	17.8% (58)	26.1% (29)	χ^2^(1) = 3.551, p = 0.059, V = 0.090
Lung and larynx (C32-34)	10.2% (33)	11.7% (13)	χ^2^(1) = 0.213, p = 0.645, V = 0.022
Female genitals / breast (C50-56)	54.8% (178)	43.2% (48)	*χ*^*2*^*(1)* = *4.403, p* = *0.036, V* = *0.100*
Male genitals (C61-62)	4.9% (16)	5.4% (6)	χ^2^(1) = 0.040, p = 0.841, V = 0.010
Leukaemia, lymphoma (C81-96)	11.4% (37)	10.8% (12)	χ^2^(1) = 0.027, p = 0.869, V = 0.008
Cancer status %* (n)*			
Tumour spread	54.5% (177)	38.7% (43)	*χ*^*2*^*(1)* = *8.182, p* = *0.004, V* = *0.137*
Lymph node metastases	20.9% (68)	22.5% (25)	χ^2^(1) = 0.126, p = 0.722, V = 0.017
Distant metastases	10.8% (35)	16.2% (18)	χ^2^(1) = 2.299, p = 0.129, V = 0.073
Relapse	3.1% (10)	0.9% (1)	χ^2^(1) = 1.593, p = 0.207, V = 0.060
Cancer treatment %* (n)*			
CT, ABT, HT	48.0% (156)	56.8% (63)	χ^2^(1) = 2.538, p = 0.111, V = 0.076
Radiotherapy	14.8% (48)	13.5% (15)	χ^2^(1) = 0.106, p = 0.745, V = 0.016
Surgery, past and present	37.2% (121)	31.5% (35)	χ^2^(1) = 1.170, p = 0.279, V = 0.052
Artificial nutrition	0.9% (3)	2.7% (3)	χ^2^(1) = 1.931, p = 0.165, V = 0.067
Rehabilitation	14.8% (48)	10.8% (12)	χ^2^(1) = 1.092, p = 0.296, V = 0.050
No treatment	19.1% (62)	23.4% (26)	χ^2^(1) = 0.970, p = 0.325, V = 0.047
Internet user (usually) *% (n)*	90.3% (290)	67.3% (72)	χ^2^*(1) = 32.699,* *p < 0.001, V = 0.276*
Health literacy score at baseline* M (SD)*	33.852 (7.699)	33.088 (8.809)	F(1, 319) = 0.518, p = 0.472, η^2^ = 0.002

The user and non-user groups differed significantly (all small effects) in the level of education (users 12.6 years vs. non-users 11.8 years), the number enrolled as inpatients (77.2% of users vs. 64.9% of non-users), the incidence of cancer of the female genitals/breast (54.8% of users vs. 43.2% of non-users) and tumor status (54.5% of users vs. 38.7% of non-users with metastatic tumor).

The difference between people who answered ‘yes’ in at least one of the follow-up surveys to the question of whether they usually use the internet to search for information was also evident. Significantly more users answered ‘yes’ than non-users (90.3% vs. 67.3%).

On average, users were aged 58 years old, predominantly female (70.2%), married (69.5%), had an acute cancer (78.4%) and were currently undergoing treatment (80.9%).

#### Patient navigators

The PIKKO study involved 15 PNs who were predominantly female (93.3%, 14/15) and had a mean age of 46 years (SD = 12.6). At the beginning of the study, they had worked in the medical sector for 20.5 years (SD = 9.7) on average, and all had a further qualification in oncology with an average of 15.4 years (SD = 8.8) of experience. Professionally, 33.3% were medical practice assistants (5/15), 60.0% were nurses (9/15), and one (6.7%) was a physician. In addition to qualifications in oncology, 13.3% had advanced qualifications in social work (2/15), 26.7% as a breast care nurse (4/15), 40.0% in palliative care (6/15), and 26.7% in pain management (4/15). 60% (9/15) worked, recruited, enrolled, and primarily met patients at a medical department of a hospital, 20.0% in a nonmedical department of a hospital (3/15), and 20.0% in an outpatient unit of an oncological specialist (3/15). During the project, three PNs dropped out, one for personal and two for health reasons.

### Utilization of WDB

As Table 3 shows, patients represented the majority (96.9%) of users, while PNs were responsible for about one-third of all accesses (33.9%). However, individual patients used the WDB on average less (twice) than the individual PN (37 times), although there were patients with a much higher usage (maximum 30 times). There was little difference in mean daily use between patients (1.1) and PNs (1.2). However, the mean total time spent using the WDB also differed between the groups and was higher for the PNs (328 min vs. 29 min for patients). This was contrasted in time spent per access, where patients stayed longer (12 min) on the WDB than PNs (9 min). Longer-term use of the WDB was only found in a few patients. Less than 10% (37/413) of patients used the WDB beyond a period of half a year, whereas the majority (12/13) of PNs did so.

**Table 3:** Characteristics of the two user groups (patients and patient navigators, PNs) with regard to different parameters for using the WDB

	Patients	PNs
Number of Users^1^	96.9% (413/426)	3.1% (13/426)
Accesses to the WDB^1^	66.1% (940/1,423)	33.9% (483/1,423)
Accesses per single user^2^	2 (2), 30, 1	37 (69), 261, 13
Accesses per day per single user^2^	1.1 (0.3), 3.0, 1.0	1.2 (0.2), 1.5, 1.2
total time spent on the WDB per individual user in minutes^2^	29 (49), 408, 12	328 (542), 2,043, 105
total time per individual user per access in minutes^2^	12 (13), 92, 8	9 (3), 15, 8
one-time accesses^1^	55.2% (228/413)	0
accesses within 1 month^1^	15.2% (63/413)	0
accesses within 3 months^1^	10.4% (43/413)	7.7% (1/13)
accesses within 6 months^1^	10.2% (42/413)	0
accesses within 9 months^1^	3.9% (16/413)	38.5% (5/13)
accesses within 12 months^1^	2.9% (12/413)	7.7% (1/13)
accesses within more than 12 months^1^	2.2% (9/413)	46.2% (6/13)

^1^absolute number and percentage, ^2^mean (standard deviation), maximum, median

### Topic analysis

**Fig. 2:**
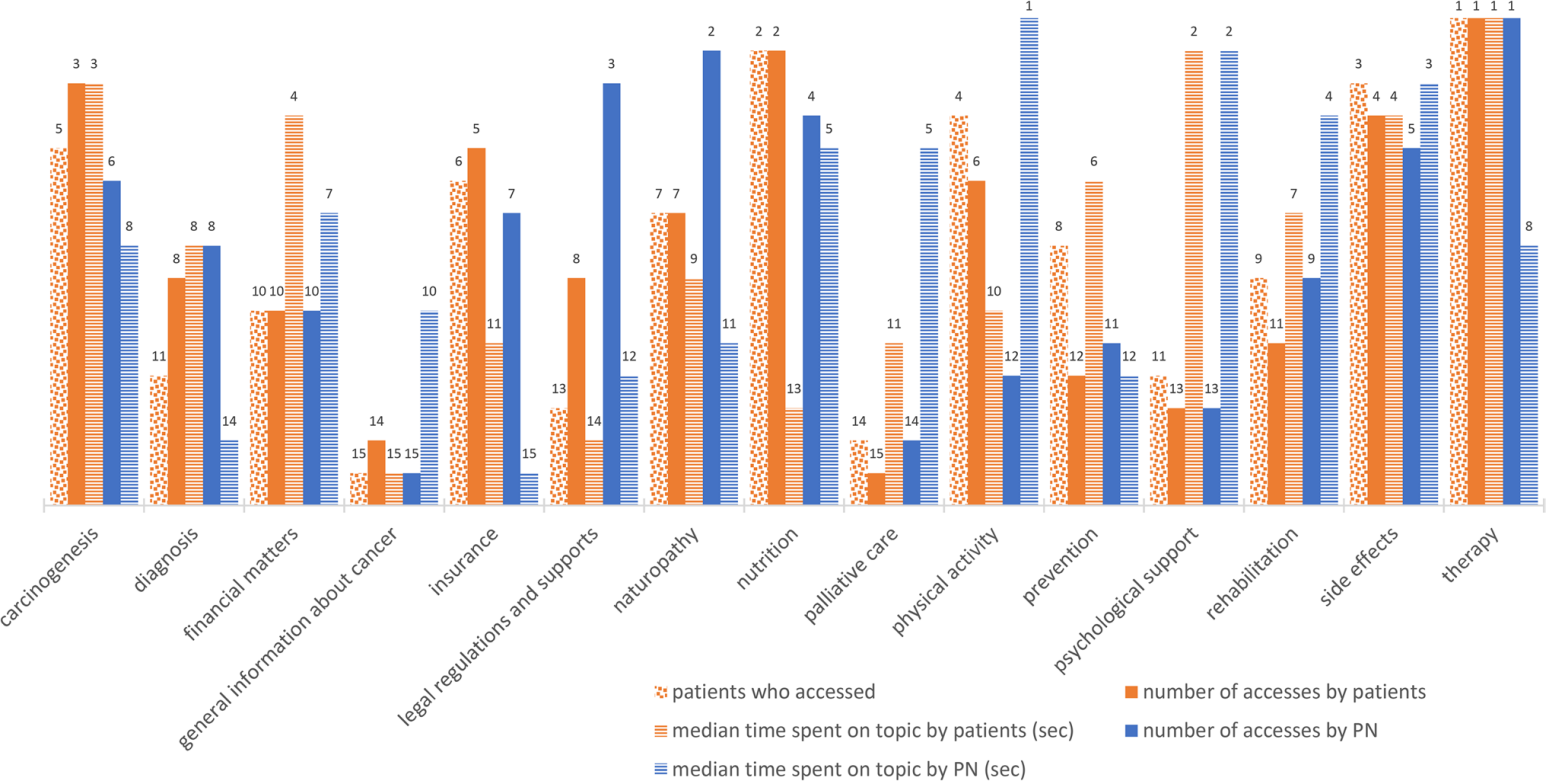
Ranking of WDB topics by number of patients accessing topics (dotted orange), by number of accesses by patients (filled in orange), by median time spent accessing patients (dashed orange), by number of accesses by PNs (filled in blue), by median time spent accessing PN (dashed blue). The time is given in seconds. Data labels indicate the respective rank within the group and thus indicate the importance of the topics (1 = most frequent, longest time to 15 = least frequent, shortest time)

The topic analysis (Table 4 and Fig. 2) examined the categories to which the pages were assigned and the frequency with which these categories were accessed, irrespective of the exact type of cancer represented by the content.

**Table 4:** Topic analysis of the two accessing groups: patients and patient navigators (PNs). Absolute number and percentage of patients who accessed the topic T(Pat), absolute number and percentage of accesses to the topic T(A), the median time spent on the topic in seconds T(t)

Topic	Patient			PNs	
	T(Pat)	T(A)	T(t)	T(A)	T(t)
therapy	41.6% (172/413)	17.4% (1,063/6,096)	98	27.6% (798/2,890)	24
nutrition	40.9% (169/413)	12.9% (785/6,096)	41	8.0% (232/2,890)	30
side effects	34.1% (141/413)	7.9% (483/6,096)	74	7.6% (219/2,890)	50
physical activity	32.0% (132/413)	6.3% (386/6,096)	52	1.4% (41/2,890)	78
carcinogenesis	31.5% (130/413)	10.1% (617/6,096)	76	7.2% (209/2,890)	24
insurance	30.0% (124/413)	7.6% (463/6,096)	51	6.5% (189/2,890)	10
naturopathy	29.1% (120/413)	6.1% (371/6,096)	55	15.3% (442/2,890)	21
prevention	22.3% (92/413)	4.3% (260/6,096)	69	1.9% (55/2,890)	13
rehabilitation	22.0% (91/413)	4.8% (291/6,096)	66	4.6% (133/2,890)	35
financial matters	20.1% (83/413)	5.0% (302/6,096)	74	3.6% (104/2,890)	28
diagnosis	18.4% (76/413)	6.1% (370/6,096)	56	4.8% (139/2,890)	12
psychological support	18.4% (76/413)	3.1% (192/6,096)	86	1.3% (39/2,890)	61
legal regulations and supports	18.2% (75/413)	6.1% (370/6,096)	31	8.5% (245/2,890)	13
palliative care	8.0% (33/413)	0.9% (52/6,096)	51	1.1% (31/2,890)	30
general information about cancer	4.6% (19/413)	1.5% (91/6,096)	21	0.5% (14/2,890)	23

Regarding the number of patients (Fig. 2, orange bars) interested in each topic, the top three topics were *therapy* (41.6%, #1), *nutrition* (40.9%, #2) and *side effects* (34.1%, #3). In terms of number of accesses by this group, ranks 1 (17.4%) and 2 (12.9%) were the same, but *carcinogenesis* held the 3rd place (10.1%) and relegated *side effects* to the 4th place (7.9%). In terms of mean time spent on topics, *nutrition* fell to 13th place (41 s), while *psychological support* took over 2nd place (86 s) (1st place remained *therapy* with 98 s, 3rd place was *carcinogenesis* with 76 s).

In the PN group (Fig. 2, blue bars), the most frequent accesses were related to pages dealing with *therapy* (27.6%, #1), *naturopathy* (15.3%, #2) and *legal regulations/support* (8.5%, #3). The top 3 differed significantly when looking at the median time PNs spend on the topics. Here, *physical activity* was #1 (78 s), *psychological support* was #2 (61 s), and *side effects* was #3 (50 s).

*Therapy* was the top topic in 4 of the 5 variables.

### Patient usage, acceptance and rating of WDB

In response to the question ‘*What was the purpose of using the WDB?’,* patients could choose one or more responses. 60.0% (195/325) used the WDB to prepare for a medical appointment, while 51.4% (167/325) used it for appointment with the PN. 83.7% (272/325) of patients accessed the WDB to answer questions regarding cancer or treatments that arose, while 92.3% (300/325) and 87.4% (284/325) of patients used the WDB to get general or specific information about cancer, respectively.

Of the 325 patients surveyed, 224 (68.9%) reported that "My PIKKO" helped them make more informed decisions. 75.7% (246/325) reported finding all the information they wanted. That "My PIKKO" was an appropriate way to provide information about diseases was affirmed by 89.8% (292/325) of patients. The 132 open-end comments from 59 patients regarding suggestions for improvement or missing information or functions elucidated that 83.1% *wanted more information* (49/59); 18.6% *suggested further functionalities* (11/59); 11.9% *wished for interactivity with medical staff* (7/59); 10.2% *desired structural, design change* (6/59); 8.5% *wished for the possibility for exchanges with other patients* (5/59); and 3.4% *wanted appointment functions* (2/59).

On a scale from 1 to 6, the WDB was given a mean overall grade of 2.16 (SD = 0.74, median = 2). This corresponds to the second best grade (‘good’) in the German school system.

### Health literacy

**Fig. 3:**
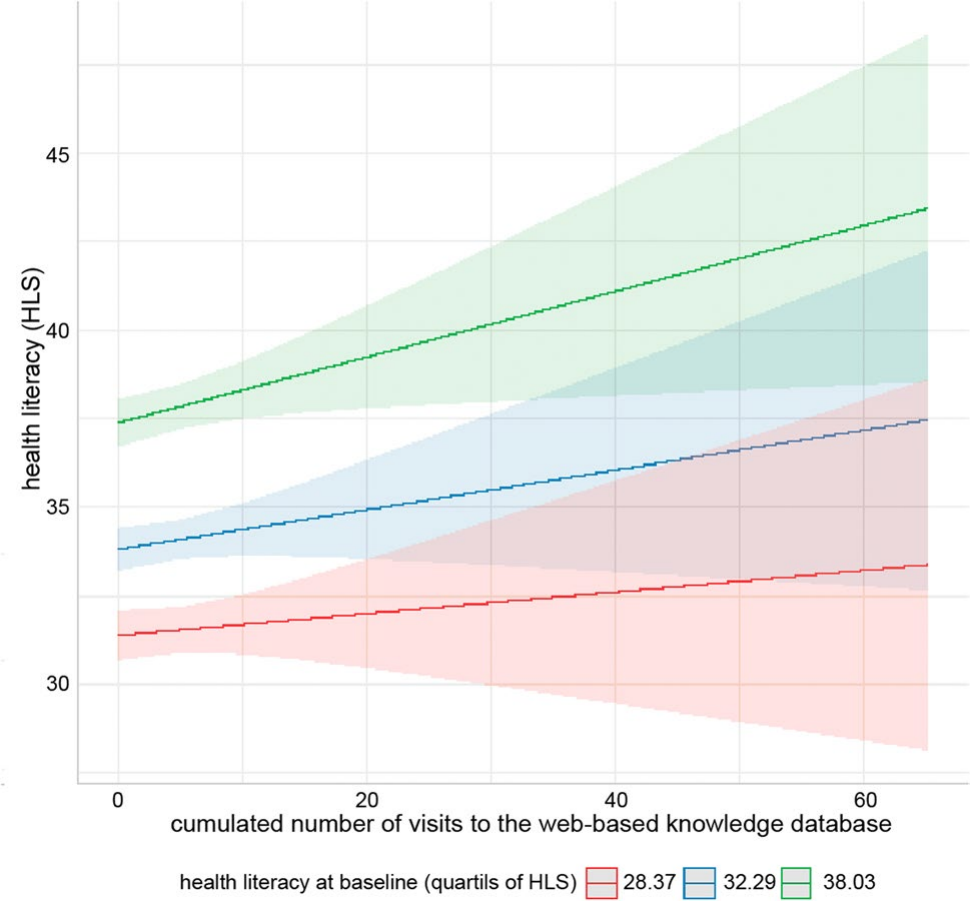
Predicted values of health literacy as a function of cumulated number of visits to the WDB and baseline health literacy. Results of growth curve models [29]

The curve growth models (Table 5) revealed an interaction effect between initial health literacy and use of the WDB [29]. Patients with initially higher health literacy benefit significantly more from more frequent use of the WDB through improved health literacy scores (Fig. 3).

**Table 5:** Regression analyses for dose effects of visits to the WDB

predictors	b	SE	*β*
intercept	13.22 ^***^	1.23	0.01
Pre-test as baseline	0.62 ^***^	0.04	0.66
t (0 = baseline)	0.20	0.17	0.02
Sessions	-0.08	0.13	0.11
Sessions x t	-0.01	0.02	-0.01
Sessions x pre-test	0.01 ^*^	0.00	0.05

^***^ p < .001, **p < .01, * p < .05, b… regression coefficient, SE… standard error, β… standardized regression coefficient

## Discussion

This study aimed to evaluate the utilization, patient ratings, and patient acceptance of a web-based database that offers evidence-based information on cancer to patients in a language understandable to laypersons.

### Key results

Who? About 66% of patients who received a login used it. Reasons for not using it were primarily technical or time-related. Only in a few cases did the patients already feel informed and therefore saw no need for it. The users reported that they in general use the internet to search for information significantly more often than the non-users. Responsible for nearly one-third of accesses over the entire period of availability, were PNs.

How? PNs used it significantly longer than individual patients. They demonstrated it to patients or prepared for a patient contact. Patients stopped using it when their questions were resolved, their cancer treatment was completed, or when they did not find what they were looking for. However, patients spent more time per access, suggesting that they engaged more deeply with the content.

What? The vast majority of patients surveyed said that they used it as a source of information, rather than to specifically prepare for appointments. Nearly 70% felt more informed by using it, and 75% found what they were looking for. Content of interest differed between the two user groups. *Therapy*, *nutrition*, *side effects* and *carcinogenesis* were the topics of high interest among patients, as well as *psychological support*, if you look at the time spent on the topics. Among PNs, some favorite patient topics (*therapy*, *side effects, psychological support*) were read often and long, but additionally *naturopathy*, *legal regulations/support*, and *physical activity* were of greater interest. These may have been topics that were requested by patients in counseling sessions, so PNs prepared for these topics.

Quality? Almost 90% of the patients surveyed considered the WDB to be a suitable tool of searching for medical information, an assessment which was also expressed by PNs in verbal conversations with us. Patients gave the overall rating of ‘*Good’*, which suggests potential for improvement. This could be seen from the open-ended comments of the surveys where patients suggested that additional more detailed or in-depth information on a wide range of topics would improve the service.

### Limitations

First, the PIKKO eligibility criteria reduced the potential users. Further, only the IG could use the WDB. Finally, not every potential user could or would use the WDB because of the reasons described above (technical, need, time, avoidance, health, motivation). Thus, the number of actual users is considerably smaller than the number of potential users within the overall PIKKO collective. A selection bias and the fact that there are multiple barriers to participate in studies [34] and to use of the internet for information [35] limit the generalization of the results, so interpretations and conclusions should always be considered with these limitations in mind.

The WDB was primarily a source of information for cancer patients. There were no interactive elements that would have provided an additional incentive to use it.

Although our two data sources referred to the same population (patients in PIKKO) the two sources could not be linked due to the anonymous data collection in the logfile. While it could be assumed that patients who provided information on the utilization of the WDB in the patient survey actually used it, this cannot be verified with absolute certainty.

### Interpretation

The digital offer of an easily accessible, quality-assured, and evidence-based information source was well received by cancer patients. Although not all participants used the WDB, the high percentage of users showed that there was sufficient interest and need. PNs also used it to prepare for appointments with patients and were thus better able to answer frequently-asked questions. Both, the usage on its own as well as the usage by PNs ultimately fulfilled the cancer patients' need for information.

Measurable benefits were seen in the fact that a majority of users felt better informed and found the information they were looking for. As we showed in further PIKKO analyses [29], patients with already high health literacy at baseline, as measured by the HSL-EU-Q47 [30] benefited in particular. These patients increased their health literacy by using the WDB (analysis of dose effects). Presumably, higher health literacy enables better reception of information (e.g. via the WDB), which in turn has a positive effect on health literacy. This fits with the finding that WDB users were also significantly more likely to use the internet as a general source of information. In another paper, we were also able to show that the WDB continued to be used in times of the first COVID-19 lockdown and that even the number of accesses increased [36].

The relatively short time spent on the pages may be related either to the fact that patient and PN questions could be clarified quickly. Missing information could be another explanation. One suggestion for improvement is therefore to expand the content. Furthermore, the WDB did not offer interactive elements that kept users on the site longer, which leads to further potential for improvement.

Apps were gaining more importance than individual websites during the last years [37]. For this reason, the technical conversion to a native app could also represent an opportunity to improve the utilization and health related effects of the WDB.

Frequently accessed topics by patients often related to the acute treatment of cancer or how to deal with it. This can be attributed to the fact that many patients with new diagnoses cancer or undergoing acute treatment were recruited in the PIKKO project. Almost exclusively, this recruitment took place in hospitals or oncology practices. In contrast, recruitment did not take place in places of aftercare such as general practitioners' practices or rehabilitation facilities. Patients in aftercare may have other topics of interest.

## Conclusion

Access to reliable information is essential for health related decision making. Our findings confirm that cancer patients have a high need for information [5] and are willing to seek it out digitally [6]. Persons who advise cancer patients (here, PNs) also benefitted from access to a source of information to better answer patients' questions. Currently, especially in the context of cancer, there exists an amount of dangerous disinformation online [38], high quality information websites are less visible in online searches [17], and profit-driven websites have lower quality [10].

In addition, since 2022, an artificial intelligence (AI) chatbot, ChatGPT 3.5, has been available to a broad, interested public [39]. This adds another dimension to information searches. The ability to have an AI search the internet for information carries the risk that without source identification, the origin of the information and if it is trustworthy cannot be known with certainty by users. Here, medical care faces new challenges in terms of patient information.

Information sources like "My PIKKO" are an essential step for improving the information situation on the internet. The WDB represents a trustworthy, evidence-based and target group-appropriate source of information for cancer patients.

### Supplementary Information

Below is the link to the electronic supplementary material.

ESM 1 (PNG 478 KB)

High Resolution Image (Two screenshots of the WDB. The main menu is shown at the top. The blue icon "Krebsarten" has been replaced by the cancer type activated for the patient. The lower area shows a subpage on the topic of therapy TIF 3889 KB)

## Data Availability

The datasets generated and/or analyzed during the current study are available from the corresponding author on reasonable request.

## Abbreviations

CG: Control group
GCS: German Cancer Society
IG: Intervention group
PIKKO: Patient information, communication and competence empowerment in oncology
PN: Patient navigator
WDB: Web-based knowledge database
