# Supplementary figures and images for: A web-based knowledge database to provide evidence-based information to cancer patients: Utilization within the PIKKO study
Source: Support Care Cancer. 2024 Jul 17;32(8):521. doi: 10.1007/s00520-024-08725-7 (PMC11254981; doi:10.1007/s00520-024-08725-7)

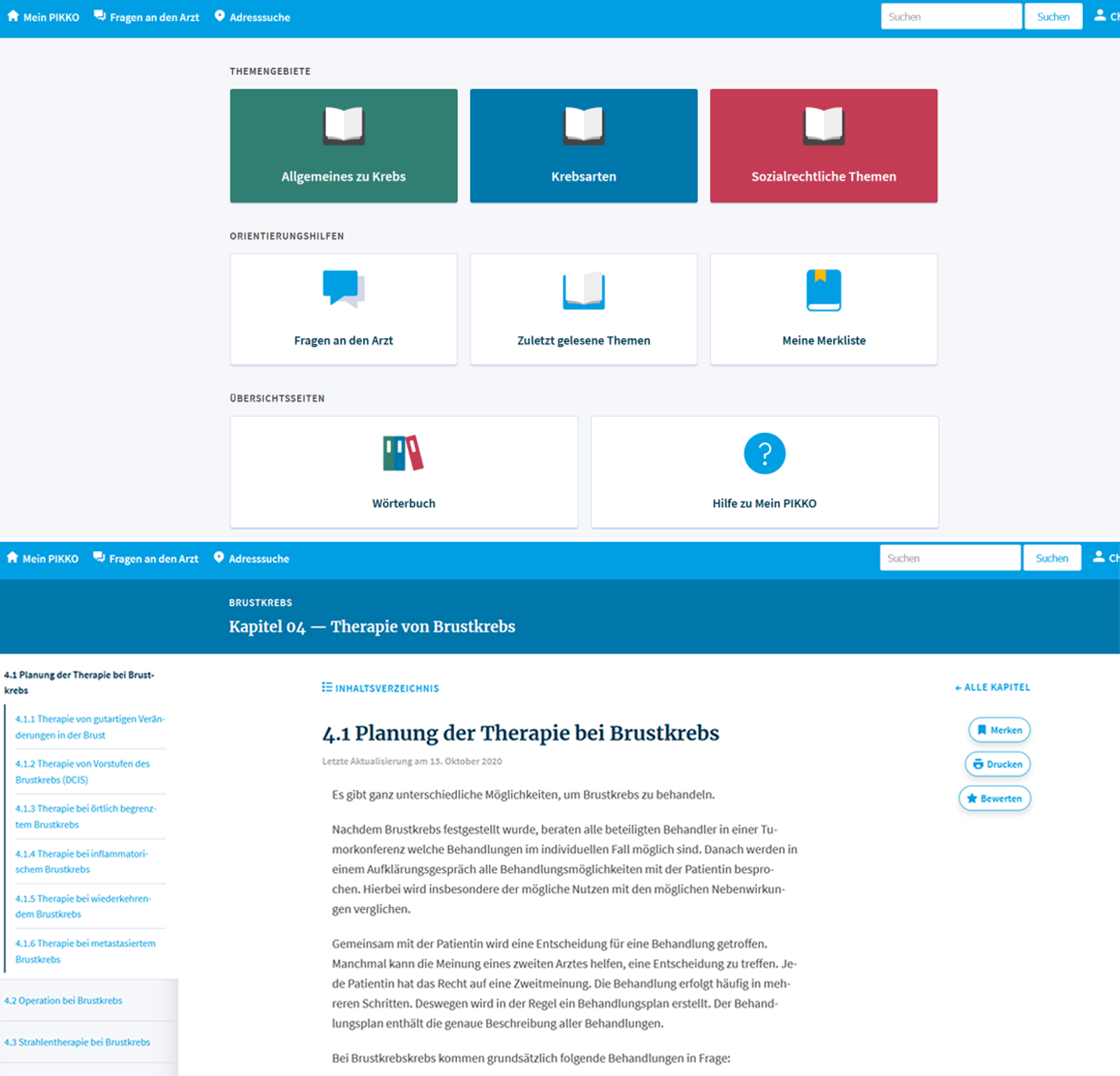

Supplement: Supplementary file 1 — (PNG 478 KB) [file 520_2024_8725_Fig4_ESM.png]

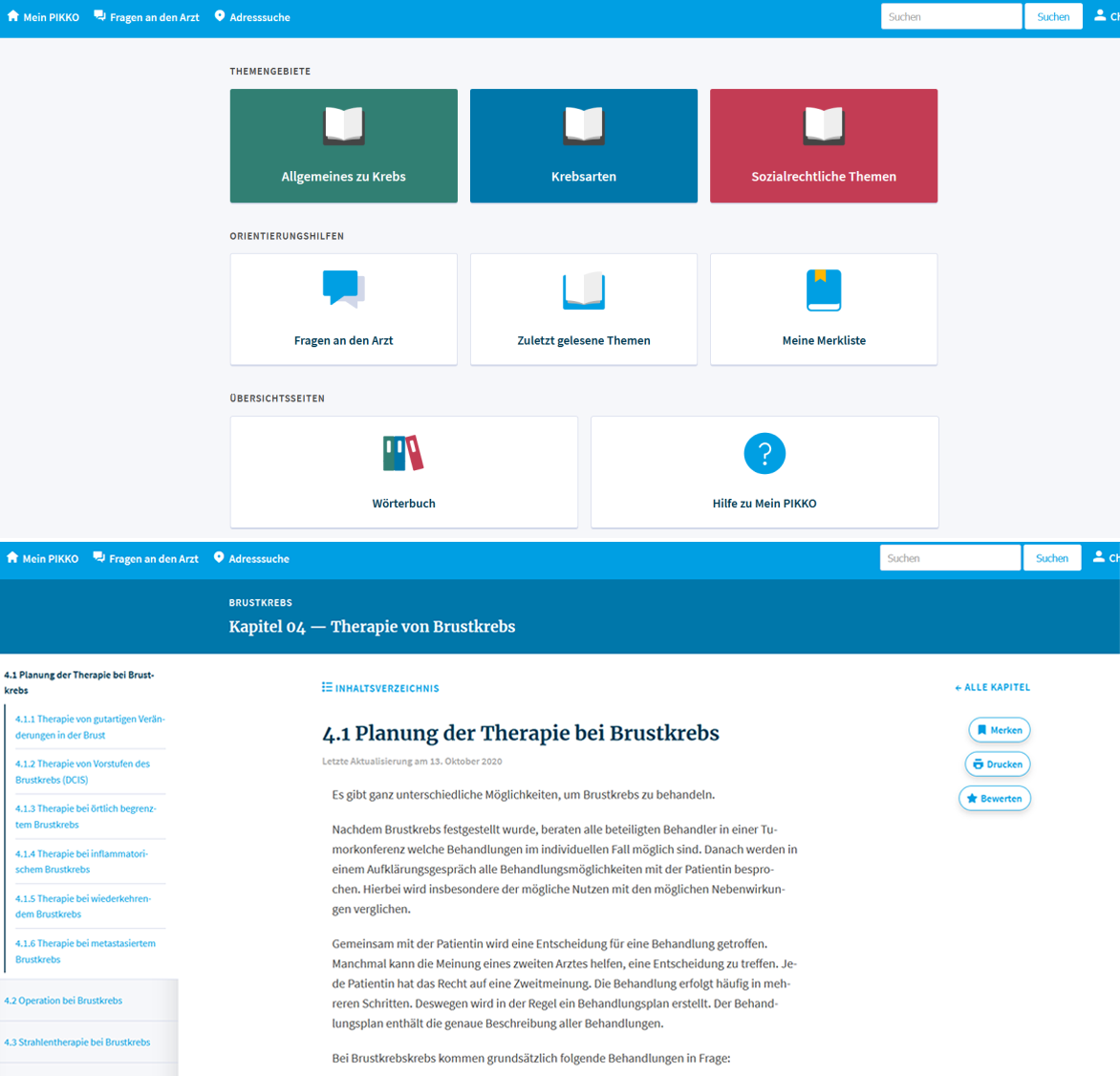

Supplement: Supplementary file 2 — High Resolution Image (Two screenshots of the WDB. The main menu is shown at the top. The blue icon "Krebsarten" has been replaced by the cancer type activated for the patient. The lower area shows a subpage on the topic of therapy TIF 3889 KB) [file 520_2024_8725_MOESM1_ESM.tif]
